# Cr-Free Anticorrosive Primers for Marine Propeller Applications

**DOI:** 10.3390/polym16030408

**Published:** 2024-02-01

**Authors:** Annie Wang, Karnika De Silva, Mark Jones, Wei Gao

**Affiliations:** 1 Department of Chemical and Materials Engineering, The University of Auckland, 20 Symonds Street, Auckland 1142, New Zealand; awan110@aucklanduni.ac.nz (A.W.); mark.jones@auckland.ac.nz (M.J.); 2 New Zealand Product Accelerator, Faculty of Engineering, The University of Auckland, Building 903, 314-390 Khyber Pass Road, Auckland 1023, New Zealand; k.desilva@auckland.ac.nz

**Keywords:** marine propellers, anticorrosion coatings, Cr-free, electrolysis, polyvinyl butyral

## Abstract

Marine propellers work under severe service conditions, where they commonly suffer from mechanical, electrochemical, and biological corrosion damage. The major mechanical corrosion involves cavitation, erosion, and impingement corrosion. On the other hand, the major electrochemical corrosion involves galvanic corrosion and electrolysis. As a result, consideration of both desired mechanical and electrochemical properties is necessary when designing a marine propeller coating. In this study, a PVB (polyvinyl butyral) and an epoxy coating were formulated without corrosion inhibitors to investigate the desired coating properties for marine propeller applications. The two coatings were compared with a Cr-containing commercial marine propeller coating to investigate the advantages and disadvantages of using PVB and epoxy for marine propeller coatings. It was found that it is desirable for marine propeller coatings to be flexible to avoid cracking and flaking; to be able to withstand high pH in order to resist cathodic disbondment (electrolysis); to have adequate primer–substrate adhesion; and, ideally, to be able to self-heal when the coating is damaged (cavitation). It was found that the PVB-ZO coating has more desirable properties, and introducing self-healing properties could be one of the options for further optimization in the future.

## 1. Introduction

Due to the surge in global international seaborne trade, the global marine vessel market is expected to increase from USD 170.75 billion in 2021 to USD 188.57 billion in 2028 [1]. This increasing demand in the marine industry means vessel maintenance and corrosion protection are essential to reduce raw material consumption, costs, and labour. Propellers, usually made of copper alloys such as bronze and brass, are the main components of the vessel propulsion system used to drive vessels forward. However, seawater is highly corrosive due to the presence of chlorine ions, which are the primary cause of the corrosion of copper alloys [2]. Although copper-based alloys have reasonably good corrosion resistance, running unprotected propellers can cause damage due to electrochemical, mechanical, and biologically influenced corrosion. Propeller failure can take different forms such as galvanic corrosion, stray-current corrosion, cavitation, erosion corrosion, and fouling. Maintenance involves mechanical or chemical removal of the corrosion or fouling products, inducing huge costs in terms of labour, raw materials, and time [3].

Applying a protective coating over the propeller surface is widely used as one of the most efficient methods to slow down propeller corrosion. Generally, the three types of anticorrosive coatings are barrier, sacrificial, and inhibitive coatings [4]. Barrier anticorrosive coatings work by physically isolating the propeller surface from the corrosive seawater. However, since there is always a certain quantity of pinholes and defects in the coating, corrosive agents may penetrate to reach the metal. Sacrificial coatings are typically zinc-rich coatings. If corrosion occurs, the zinc particles act as the sacrificial anode to protect the propeller (cathode) by oxidizing to zinc oxide, which will further deposit on the corroded area, transforming the protection mechanism from sacrificial to barrier protection [4]. 

On the other hand, an inhibitive coating protects the propeller by forming a passive protective layer over the propeller surface. Hexavalent chromium-containing coatings are a typical example and have extraordinary performance due to their ‘self-healing’ ability [5]. This means that the coating can ‘self-repair’ by forming a passive layer over the exposed metal surface to shield the corrosive ions away from the exposed substrate after the coating is damaged. However, restrictions have been placed on the industrial usage of these types of coatings since they are harmful to human health and can cause environmental pollution [6]. Therefore, the marine industry urgently needs an alternative environmentally friendly coating system with better or similar performance. 

A propeller coating system generally includes a primer and a topcoat. The primer plays a crucial role in providing anticorrosive properties and enhancing topcoat–primer and primer–substrate adhesion. The topcoat primarily prevents the propeller from fouling, resists cavitation damage, and protects the primer. The propeller surface pre-treatment is necessary prior to the application of the primer. Most published studies focus on studying the topcoat’s foul-release property and its resistance to cavitation damage. In contrast, there is little research on the anticorrosive properties of primers.

**Figure 1:**
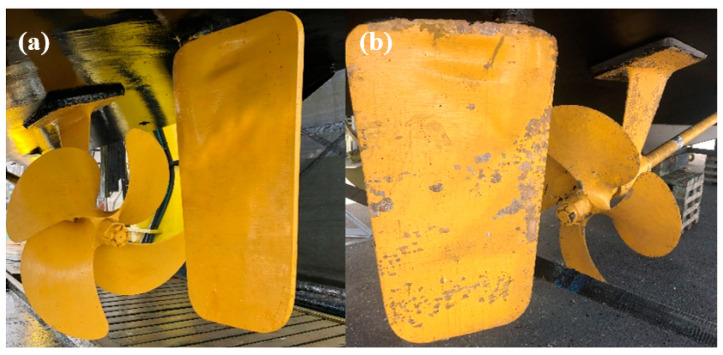
(**a**) Commercial primer applied just before launching, (**b**) after 16 months of field trial.

Several commercial products are available on the market, developed by leading marine paint companies such as Propspeed, International, and Hempel. Figure 1 shows the results of a commercial coating system after 16 months of field trial. The coating system was developed explicitly for propellers. Figure 1b shows that the commercial coating system has excellent performance even after 16 months of testing. Although the coating system suffered damage in the cavitation-prone area, most parts of the coating showed excellent adhesion to the underwater running gear. 

In addition to commercial anticorrosive propeller primers, various novel coatings have been developed over the past decades as replacements for Cr-containing coatings. These coatings include sol-gel, polymer nanocomposite, conducting polymer, and smart coatings. Lakshmi et al. developed a silica-alumina-based hybrid sol-gel coating with cerium oxide nanofibers to replace the Cr conversion coating (CCC) for an AA2024 alloy [7]. Neutral salt spray results showed that the coating performed similarly to CCC after 336 h. Ma et al. synthesized graphene oxide-cerium oxide hybrid epoxy composite coatings for AA2024 alloy protection [8]. Their research showed that the mechanical properties of the composite had been greatly improved due to the uniform dispersion of GO-CeO_2_ particles in the epoxy matrix. The corrosion resistance was also improved due to the synergies of the barrier enhancement by the GO and the inhibition effect of the CeO_2_ particles.

Niraitiwongkorn et al. synthesized a polyvinyl butyral (PVB)-based polypyrrole–carbon black composite [9]. It was reported that the coating has enhanced passivation ability and barrier properties, significantly improving the corrosion resistance of the composite coating. Jia et al. prepared a pH-responsive L-histidine loaded in graphene/halloysite nanotubes and embedded them in a waterborne epoxy matrix [10]. The self-healing coating provided multifunctional anticorrosion protection based on the excellent barrier property enhanced by HNTs-rGO and the self-healing ability induced by L-His. However, these novel coatings were not developed specifically for propeller primers. As a result, both electrochemical and mechanical property tests of coatings were not carried out, which are essential for propeller coatings to cope with the complex propeller failure forms.

Anticorrosive coating studies have primarily been focused on enhancing epoxy-based coatings [11], which are often evaluated under static conditions, such as atmospheric corrosion [12] and constant static immersion corrosion [13]. Barrier properties tend to be more critical than mechanical properties under such conditions. However, the dynamic conditions of these types of barrier coatings are rarely studied for marine propellers. Therefore, it is crucial to investigate whether the epoxy-based coatings can withstand these harsh conditions whether they are able to protect propellers. While PVB has been used for laminated safety glass for many years [14], its use in corrosion protection and organic coating formulation is a more recent development. PVB-based coatings are gaining popularity due to their excellent adhesion to the substrate, toughness [15], film forming, high tensile strength, and impact resistance properties [16]. They are softer but more flexible than epoxy-based coatings, making them more resilient to cavitation damage. Therefore, PVB coatings could potentially provide better resistance to cavitation compared to epoxy-based coatings.

Given the complex failure modes of propellers, it is believed that designing an effective propeller coating requires multidisciplinary knowledge to improve both the electrochemical corrosion resistance and mechanically induced corrosion, such as cavitation and erosion corrosion. Therefore, this paper aims to investigate the essential properties required for designing propeller anticorrosive coatings and determine which type of coating system (epoxy or PVB) is more suitable to be used as propeller coatings. To achieve this, a series of electrochemical and mechanical tests were conducted to study the potential failure mechanisms of the coatings. Additionally, mechanical tests were performed after sample immersion in salt water to assess any changes in the mechanical properties of the coatings. 

## 2. Experimental Section

### 2.1. Materials

The nature of the primers used is summarized in Table 1. These primers are solvent based. PVB-ZC is a two-component (2K) system consisting of a PVB resin and a phosphoric acid hardener, which activates zinc chromate just before use. On the other hand, a PVB-ZO primer is one of the alternatives to be tested. It is a one-component (1K) coating system with the same composition as PVB-ZC, except that zinc chromate is replaced with zinc oxide. These two PVB-based primers have equivalent pigment volume concentration (PVC) of zinc chromate and zinc oxide. Lastly, the epoxy primer (EP) is a 2K epoxy-based coating and it is a generic primer without soluble pigments. 

### 2.2. Sample Preparation

Table 2 provides a summary of the substrate used in each test. Prior to primer application, the substrate surface was thoroughly cleaned by washing with water and wet sanding with #400 grit sandpaper. The surface was dried with a clean cloth and degreased with isopropanol before being air dried and made ready for the primer application. All primers were applied using a 100 µm wire wound bar except that the electrolysis samples, for which the primers were applied at their recommended dry film thickness (DFT) to simulate the field-testing conditions. To achieve a near-identical dry film thickness (DFT) for each test, multiple coats were applied on the substrates, with each coat being applied as soon as the previous one became touch dry. This approach was used to minimize internal stress build-up that can occur within the coating when applied in a single thickness. After 7 days of curing, 20 DFT measurements were taken randomly across the coated panels (avoided panel edges) using a dry film thickness gauge (Elcometer 456, SYNTECH, Auckland, New Zealand). It should be noted that the coating thickness of the electrolysis test samples may not be as uniform as those applied with the wire wound bar, due to their preparation by brushing. The DFT of all the samples prepared for each specific test are summarized in Table 3. 

### 2.3. Electrochemical Characterisation

The electrochemical impedance spectroscopy (EIS) results were obtained by using an electrochemical workstation (CHI 650E, Shanghai, China). The setup involved a three-electrode system and an AC current with a range of 0.1 to 100 kHz and an applied amplitude of 50 mV. The samples were immersed in 3.5 wt.% NaCl and continuously monitored for 100 days. For the damaged EIS test, a horizontal artificial scratch (2 mm) was made on the coating through the substrate by using an art knife. Measurements were taken until the open circuit potential (OCP) reached a constant value. 

The potentiodynamic polarization (PDP) test was also conducted using the same electrochemical workstation (CHI 650E, Shanghai, China) and the scanning voltage was ±250 mV based on OCP. To assess the electrolytic resistance of the coating, an in-house electrolysis tank was built, and the electrolyte solution was made using synthetic sea salt (Instant Ocean^®^ Sea Salt, Blacksburg, VA, USA) at pH 7. Since the coatings were applied by brush to their recommended DFT, their DFT measurement after 7 days showed some variance. To accelerate the corrosion reaction, an artificial vertical scratch was made into the middle of each of the primer layers through to the substrate. Gorilla tape^®^ was used to insulate the edges of the panel and prevent any edge effects. The coated bronze panel and an Al anode were immersed in the electrolyte and connected to an external DC power supply. The voltage setup was 1.25 V, and the test was carried out for 24 h.

### 2.4. Mechanical Characterisation

Microhardness tests were conducted using a microhardness hardness tester (DuraScan 80 G5, ZwickRoell, Germany) with a Vickers indenter. A 10 g indentation load was applied with a dwell time of 10 s. Five measurement points were randomly selected across the coated panel surface. To minimize the substrate effect, the microhardness samples were prepared at a DFT of approximately 300 µm. A conical mandrel test apparatus was used as per the ASTM standard. Vertical scratches were made into the coated panel at 2 cm intervals to assess the cracking resistance and flexibility of the primers. Three replicates were carried out for each primer. Pull-off adhesion tests were carried out using a pull-off adhesion tester (Elcometer 506, SYNTECH, New Zealand). The aluminium dollies were sanded and cleaned with isopropanol. Cyanoacrylate glue (Selleys^®^ QuickFix™, Auckland, New Zealand) was applied to the dolly surface and then attached to the coating surface. After 7 days, the dollies were pulled using the pull-off adhesion tester and the coatings were inspected to determine whether there was adhesive or cohesive failure.

## 3. Results

### 3.1. Electrochemical Impedance Spectroscopy (EIS)

**Figure 2:**
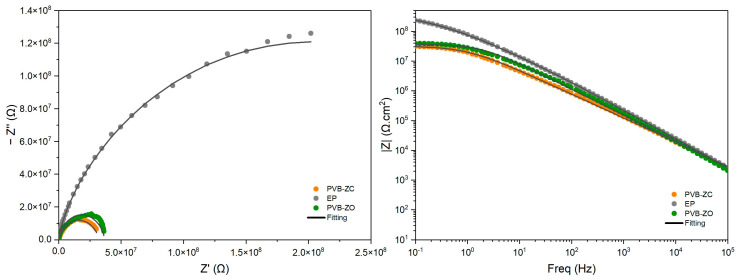
EIS results after 30 min of immersion in 3.5 wt.% NaCl. Nyquist plot (**left**) and Bode plot (**right**).

**Figure 3:**
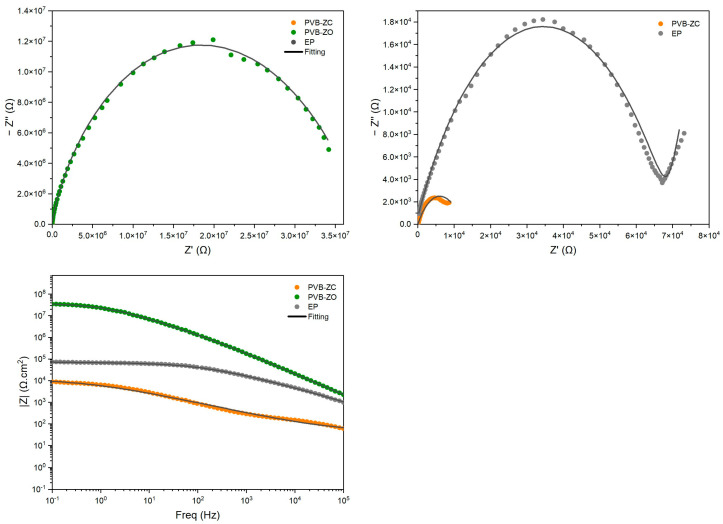
EIS results after 100 days of immersion in 3.5 wt.% NaCl. Nyquist plot (**left**), zoomed-in Nyquist plot (**right**), and Bode plot (**left bottom**).

The EIS test is a widely used technique to monitor the degradation behaviour of coatings over time. Being a non-destructive technique, one advantage of using EIS is that it is able to detect early stages of electrolyte penetration even when there are no visible changes in the coating [17]. It measures the change in interfacial properties, such as the coating impedance, which is the ability of a material to resist the flow of an AC current. In other words, EIS provides information on the barrier properties of the coating since the impedance decreases when water is absorbed by the coating. EIS is also a useful tool for predicting the corrosion resistance and service life of organic coatings applied to metallic surfaces. In this study, each primer was subjected to EIS testing for up to 100 days in 3.5 wt.% NaCl solution. The obtained Nyquist and Bode plots were analysed and are presented below (Figure 2 and Figure 3).

After 30 min of immersion, it was observed that EP exhibited the highest barrier performance, whereas PVB-ZC had the lowest barrier performance. A direct comparison of the semicircle diameters from the Nyquist plot (Figure 2) provided a quick assessment of the barrier properties of the primers. This comparison was also reflected in the Bode plot, where the impedance modulus of EP at |*Z*|_0.1Hz_ was the highest and, for PVB-ZC, it was the lowest. |*Z*|_0.1Hz_ is a useful parameter for determining the protective ability of the primers, as more information about the coating performance can be found in this low-frequency range. The |*Z*|_0.1Hz_ value in the low-frequency region decreases over time as the coating absorbs and becomes saturated with the electrolyte. As the coating starts absorbing the electrolyte solution, it shows both capacitive and resistive behaviour in the Bode plots [18,19]. Typically, if a coating has a |*Z*|_0.1Hz_ ≥ 10^6^ Ω·cm^2^, this gives an indication that the coating provides an adequate level of corrosion protection [18]. 

After 100 days (Figure 3), it was observed that PVB-ZO had the highest barrier properties and adequate corrosion protection, while PVB-ZC exhibited the lowest performance, as was the case at 30 min. Therefore, it can be concluded that PVB-ZC is more permeable than the other two primers, and that PVB-ZO has more resistance to water penetration, maintaining its barrier properties. By examining the Nyquist plot of EP at 100 days, it is noted that there is an inclined line at approx. 45° in the low-frequency region. This is the Warburg diffusion, depicting the diffusion process at the coating–metal interface [18]. Although EP has a higher crosslinking density and a higher |*Z*|_0.1Hz_ at the beginning, the value gradually decreased, meaning that the coating absorbs the electrolyte continuously.

The Bode plot clearly indicates that PVB-ZO maintained almost constant |*Z*|_0.1Hz_ values before and after 100 days of immersion, demonstrating its excellent barrier properties. Conversely, PVB-ZC has the largest decrease in |*Z*|_0.1Hz_ values, suggesting its corrosion protection mechanism is inhibitive rather than barrier based. This indicates that a certain degree of porosity is necessary for PVB-ZC to allow CrO_4_^2−^ ions to diffuse and travel easily in the coating matrix, ultimately passivating the exposed substrate. EP, on the other hand, showed a near-constant decrease in |*Z*|_0.1Hz_ values over time without any increases. This may be attributed to EP’s reliance purely on barrier protection without any anticorrosive pigments, implying that corrosion will initiate as soon as water penetrates the coating and reaches the substrate. Without any anticorrosive pigments to slow down corrosion, the barrier performance of EP will continuously decrease until the coating fails completely.

### 3.2. Artificially Scratched EIS

The intention to perform artificially scratched EIS testing is based on the initial results of the EIS tests. The purpose of the scratched EIS test is to test the protective (inhibitive) properties of the coating rather than testing its barrier properties [20]. It was initially hypothesized that PVB-ZC would exhibit the best EIS results due to its known passivation ability. However, the results showed the opposite trend, which could be attributed to the formulation of the primer, making it more porous than the other two primers. In the absence of damage to the primer that exposes the substrate, CrO_4_^2−^ ions are unable to play their role beyond diffusion/leakage into the electrolyte, leading to the electrolyte turning into a yellow colour. As a result, it was anticipated that conducting artificially scratched EIS tests would reveal the passivation/self-healing ability of PVB-ZC over the other two primers. 

**Figure 4:**
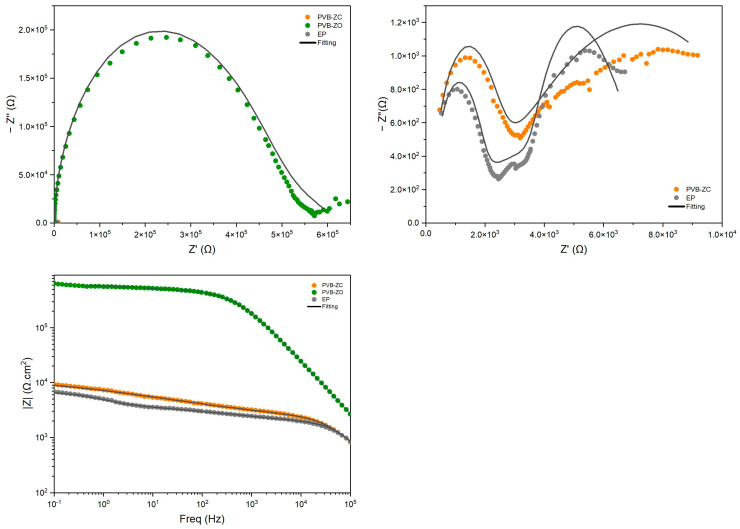
Damaged EIS results after 30 min Nyquist plot (**left**), zoomed-in Nyquist plot (**right**), and Bode plot (**left bottom**).

**Figure 5:**
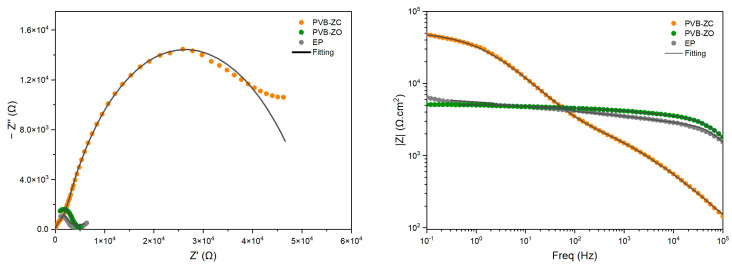
Damaged EIS results after 100 days. Bode plot (**left**) and Nyquist plot (**right**).

Figure 4 illustrates the barrier properties of the coatings at 30 min of immersion. PVB-ZO demonstrates the highest barrier performance, while PVB-ZC and EP exhibit similar performance. However, PVB-ZC has the highest barrier performance after 100 days (Figure 5) and the |*Z*|_0.1Hz_ value increases from 10^4^ to 10^4.7^. In contrast, both PVB-ZO and EP experience a continuous decrease in barrier properties, reaching a similar level after 100 days. This can be due to the absence of active corrosion inhibitors in their formulations, which would typically form a protective layer over the exposed metal surface. However, PVB-ZO incorporates ZnO in its formulation, enhancing the primer’s toughness and water resistance [21].

Initially, PVB-ZO exhibited higher barrier performance than EP. However, the key difference is that PVB-ZO experienced a more significant decrease, while EP maintained its barrier properties to a greater degree. This discrepancy may be attributed to the solubility of ZnO in dilute acids and bases [22]. As a result, ZnO dissolves at the cathodic sites under alkaline conditions (high pH), leading to a reduction in barrier properties. While ZnO has been reported to possess anticorrosive properties [21], these were not observed in the damaged EIS test. 

In contrast, PVB-ZC incorporates zinc chromate as a corrosion inhibitor. Consequently, when the substrate is exposed to the electrolyte, it undergoes passivation, leading to an increase in |*Z*|_0.1Hz_ over time. Although corrosion under the coating was assessed by the formation of the two semicircles at 30 min (Figure 4), there was only a single Nyquist semicircle after passivation at 100 days (Figure 5). Therefore, the artificially scratched EIS test proves to be more suitable for evaluating the passivation ability of PVB-ZC and other primers that exhibit self-healing properties. 

### 3.3. Potentiodynamic Polarization Test (PDP)

**Figure 6:**
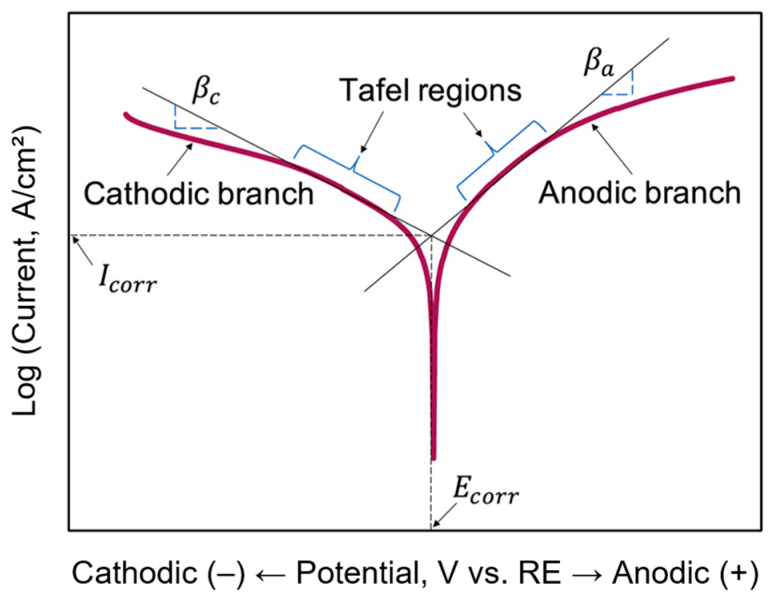
Analysis of a typical Tafel plot. Reproduced from ref. [18], copyright (2017), with permission from John Wiley & Sons Inc.

The potentiodynamic polarization test is another electrochemical test carried out in this study, which gives a better understanding of the corrosion process by measuring the thermodynamics and kinetics of corrosion. Given that the corrosion process involves both cathodic (i.e., oxygen reduction reaction) and anodic reactions (i.e., metal oxidation), which can be accelerated by scanning the sample over a narrow potential range while monitoring the current. By extrapolating the cathodic and anodic slopes in the linear regions of the Tafel plot, the corrosion potential (*E_corr_*) and corrosion current density (*I_corr_*) can be determined (Figure 6). As a result, PDP tests can provide insights into the susceptibility of coated metals to corrosion and predict the long-term damage effects. *E_corr_* indicates the level of protective ability of the coating towards the substrate, while *I_corr_* signifies the rate of electrolyte penetration into the coating. Usually, a high *E_corr_* value and a low *I_corr_* value is preferred for good corrosion resistance, which indicates less corrosion tendency and improved corrosion resistance, and a delay in the electrolyte uptake at the coating–metal interface, respectively [18].

**Figure 7:**
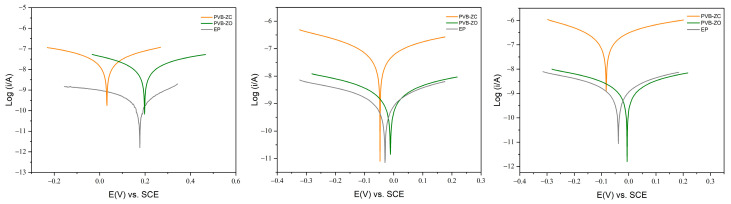
Potentiodynamic polarization test results at 3 h, 50 days to 100 days (**left** to **right**).

**Figure 8:**
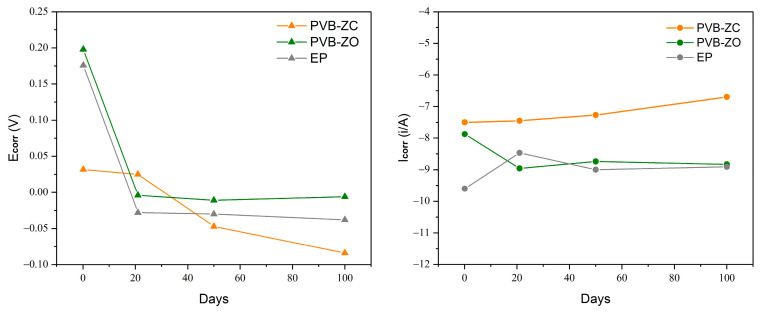
Changes in *E_corr_* (**left**) and *I_corr_* (**right**) over time.

The corrosion kinetics of the three primers were examined over a 100-day period (Figure 7), and the corresponding changes in *E_corr_* and *I_corr_* over time are presented in Figure 8. It was observed that PVB-ZC exhibited a continuous decrease in *E_corr_* over time, while PVB-ZO and EP maintained a relatively constant *E_corr_*. Similarly, PVB-ZC showed a continuous increase in *I_corr_*, whereas PVB-ZO and EP maintained a relatively stable *I_corr_*. A high *E_corr_* is preferable as it indicates that the sample is more likely to accept electrons, favouring reduction reactions to protect the substrate. On the other hand, a lower *I_corr_* is desirable as it suggests retarding of the electrolyte diffusion through the coating–metal interface. According to this theory, it can be concluded that PVB-ZC gradually loses its barrier protection over time, leading to an increased rate of electrolyte penetration. This finding aligns with the results obtained from the non-scratched EIS analysis, where PVB-ZC exhibited lower barrier protection and higher porosity compared to the other primers. This discrepancy arises from the fact that PVB-ZO and EP primarily rely on barrier protection mechanisms, while PVB-ZC is based on inhibitive protection mechanisms.

The changes in *E_corr_* and *I_corr_* also showed that PVB-ZO and EP are mostly based on barrier protection mechanisms as the behaviour of the changes are similar (also they do not have any active corrosion inhibitive additives in their formulation). The PDP test also shows that ZnO does not demonstrate any anticorrosive properties but barrier enhancement. The PDP results at Day 100 also align with the scratched EIS results, where the final *I_corr_* values are similar for PVB-ZO and EP, and *E_corr_* are also closer together. The similar performance between EP and PVB-ZO after 100 days of immersion are demonstrated both by EIS and PDP test.

### 3.4. Electrolysis Corrosion

**Figure 9:**
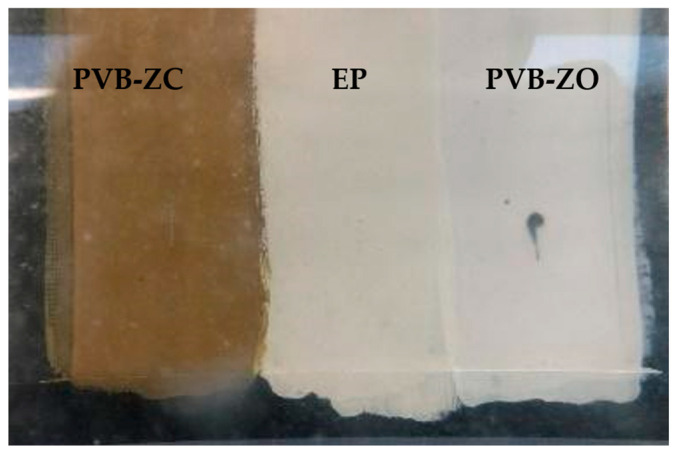
Appearance of the coated bronze plate after 30 s of immersion in the electrolysis tank.

Electrolysis or stray current corrosion is a common type of corrosion that occurs on immersed marine propellers. In freshwater environments, electrolysis may not be as severe as in seawater, where the high conductivity due to chlorine ions exacerbates the corrosion process. Figure 9 illustrates the appearance of the panel immediately after the test commenced. The area surrounding the PVB-ZO scratch turned black within just 30 s of immersion. In contrast, no evident damage was observed on the other two primers, although bubbles were observed from the scratched area for all three primers, possibly indicating the formation of hydrogen gas.

**Figure 10:**
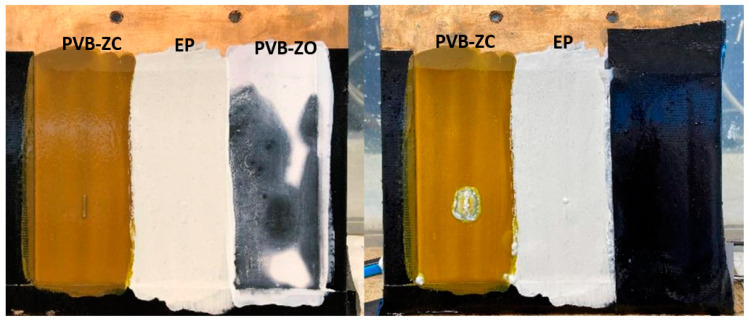
Appearance of the coated plate after 20 min (**left**) and 24 h (**right**) of testing.

After 20 min (Figure 10), the blackened area on the PVB-ZO primer continued to expand across the coated region, and appeared to detach from the substrate, indicating potential cathodic disbondment. At this point, PVB-ZC and EP remained in good condition. After 40 min, PVB-ZO had completely failed, resulting in the removal of the black film. Some damage was observed on the PVB-ZC primer, although the region around the scratch did not peel off but instead exhibited a silverish colour on the substrate. EP, on the other hand, demonstrated the highest resistance to electrolysis, as no coating peeling or dissolution was observed. However, the observed performance of EP could be attributed to its recommended DFT being 200 µm whilst the other coatings were only 16 µm and 50 µm for PVB-ZC and PVB-ZO, respectively.

### 3.5. Microhardness Test

Microhardness gives a valuable measure of a primers’ resistance to surface deformation and impingement [23], and provides insights into the crosslinking density of the coating. Table 4 shows the microhardness before immersion, showing that the PVB-based systems (thermoplastic) exhibit lower hardness compared to the epoxy-based systems (thermoset). This discrepancy indicates that epoxy primers possess a significantly higher crosslinking density, aligning with the film-forming mechanism inherent to each coating system.

**Figure 11:**
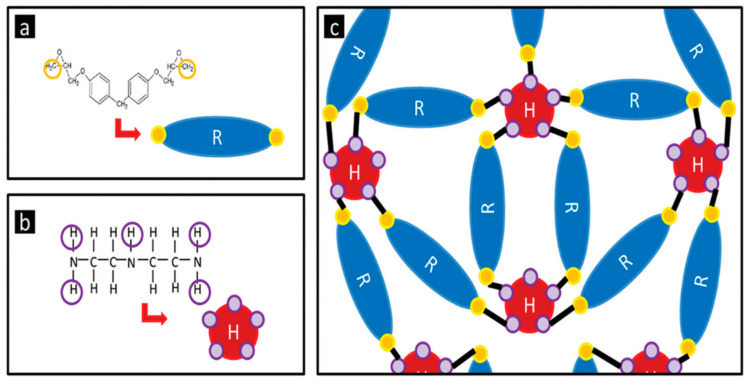
Molecular structure of an epoxy resin with two active sites (**a**), a polyamine curing agent with five active sites (**b**), and the crosslinked structure (**c**). The black solid lines represent the covalent bonds. Reproduced from [24], copyright (2015), with permission from Sage Journals.

Among the primers, PVB-ZO exhibits the lowest hardness due to its film formation process, which relies solely on solvent evaporation. On the other hand, PVB-ZC is harder than PVB-ZO but softer than EP. EP has a much higher microhardness due to a different film-forming mechanism. Epoxy resin requires a hardener to induce the crosslinking density to form a solid film. The hardener opens the epoxide ring and forms crosslinks across the chemical structure (Figure 11). This increase in the crosslinking density of the coating matrix results in a higher microhardness for EP than the other two coatings. 

### 3.6. Conical Mandrel Bend Tests

The conical mandrel bend test was conducted to evaluate the cracking resistance of the primers. Vertical straight scratches spaced 2 cm apart were intentionally made on each panel. This approach allows for the assessment of primer adhesion and flexibility in proximity to areas prone to cracking. The test was repeated after immersing the panels in 3.5 wt.% NaCl for 80 days.

**Figure 12:**
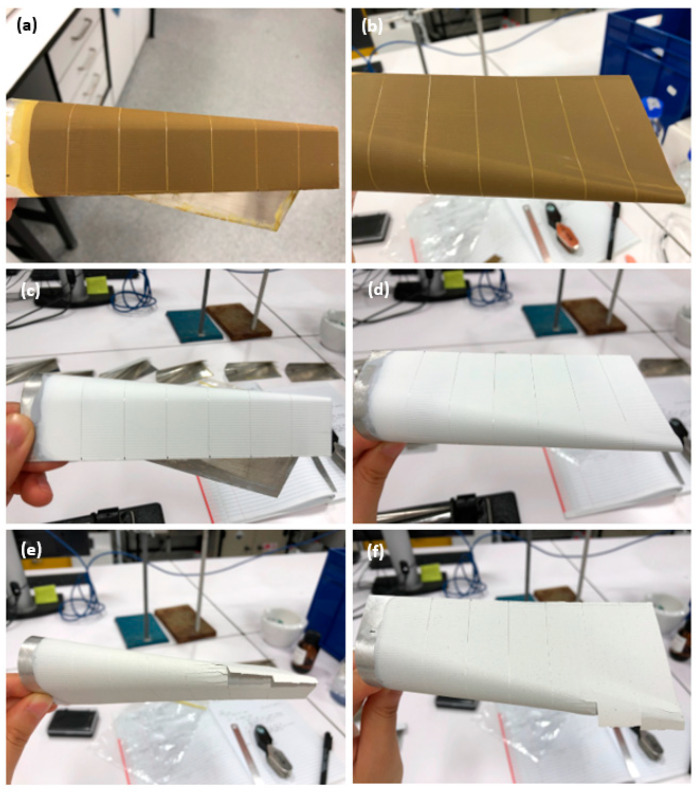
Conical mandrel bend results before immersion: (**a**,**b**) PVB-ZC, (**c**,**d**) PVB-ZO, and (**e**,**f**) EP.

Figure 12 illustrates the results of the conical mandrel bend test. Notably, PVB-ZC exhibited no cracking even in the vicinity of vertical artificial scratches, demonstrating excellent flexibility. Similarly, PVB-ZO displayed good flexibility during bending, showing no signs of cracking. However, EP exhibited cracking upon bending, and two out of three replicates exhibited peeling from the substrate. Furthermore, a section of EP at the small end of the conical diameter detached completely.

**Figure 13:**
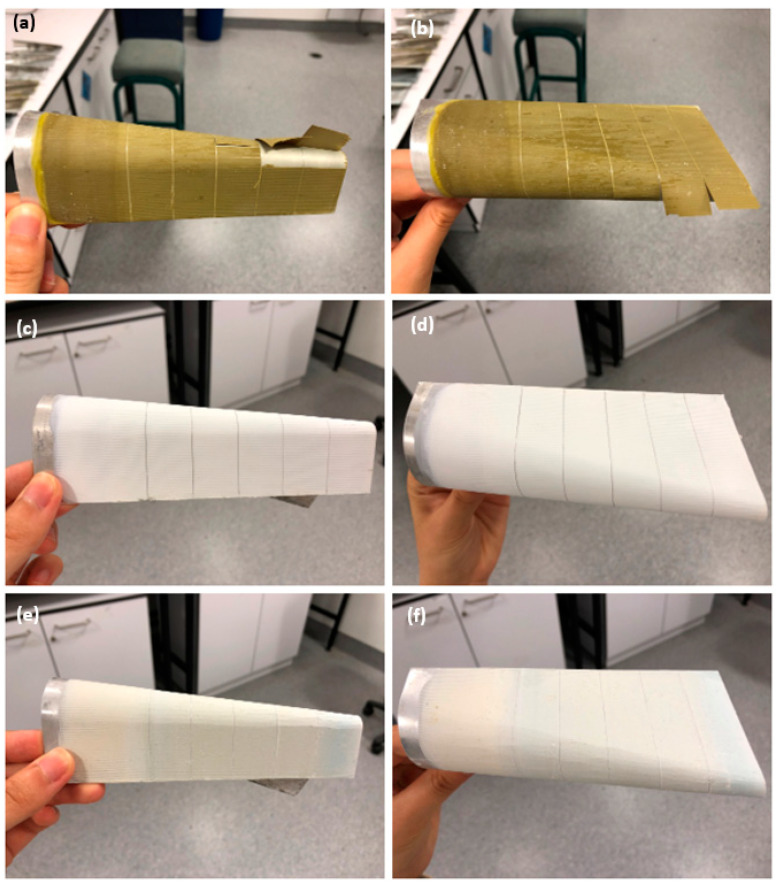
Conical mandrel bend results after 80 days immersion in 3.5 wt.% NaCl: (**a**,**b**) PVB-ZC, (**c**,**d**) PVB-ZO, and (**e**,**f**) EP.

However, the results took a different turn after immersing the samples in 3.5 wt.% NaCl for 80 days. The PVB-based systems performed differently, becoming more brittle following immersion as shown in Figure 13. PVB-ZC exhibited noticeable cracking and peeling from the substrate, indicating decreased flexibility. Similarly, PVB-ZO displayed cracking around the small cylinder diameter at one end, although no peeling was found. On the other hand, all replicates of the EP showed improved flexibility after immersion. While some cracking was still present, its flexibility was increased compared to the pre-immersion condition, and no peeling was observed.

### 3.7. Pull-Off Adhesion

**Figure 14:**
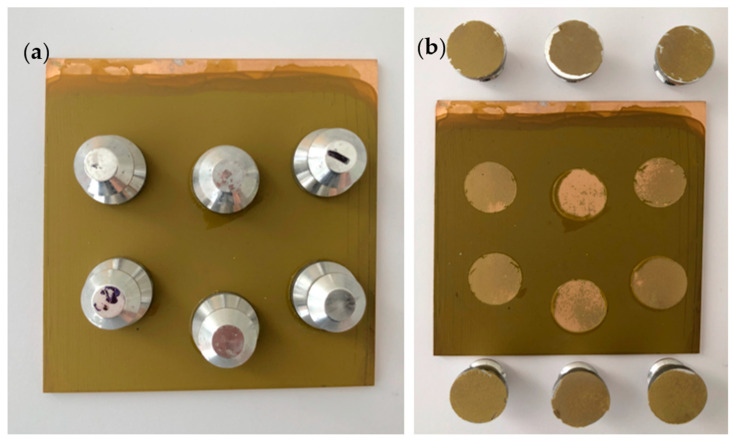
The pull-off adhesion test of PVB-ZC before (**a**) and after (**b**) pulling.

**Figure 15:**
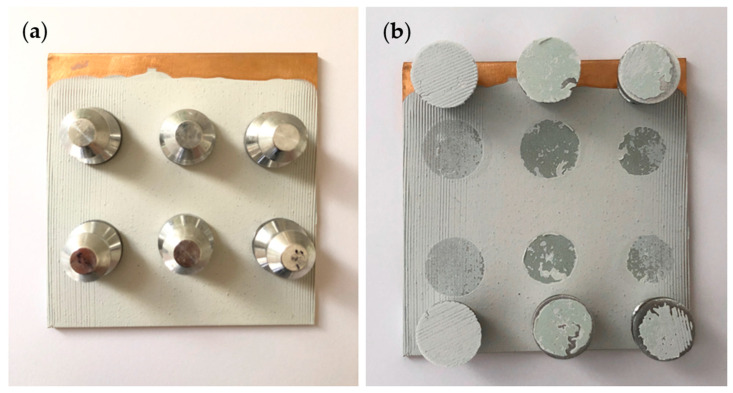
The pull-off adhesion test of EP before (**a**) and after (**b**) pulling.

**Figure 16:**
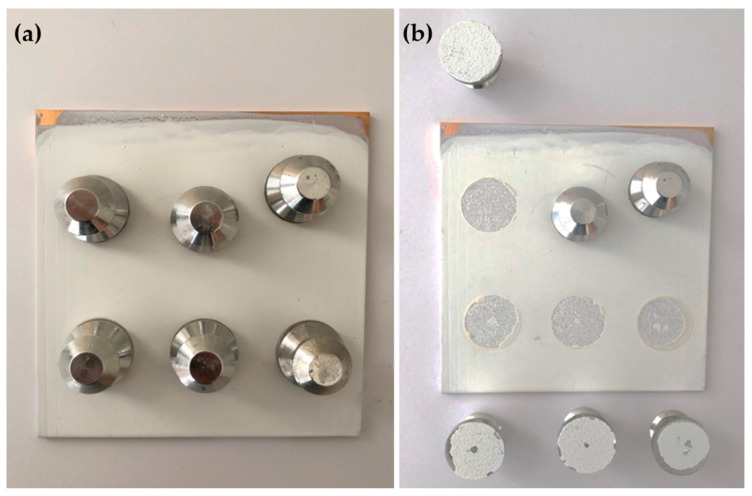
The pull-off adhesion test of PVB-ZO before (**a**) and after (**b**) pulling.

The pull-off adhesion test was conducted to evaluate the adhesion of primer to the substrate. The fracture interface was examined to determine whether failure was adhesive or cohesive in nature. Adhesive failure indicates the coating failure occurs at the coating-metal interface, while cohesive failure suggests the failure occurs within the coating. Adhesion is a crucial property/prerequisite for primer performance, as even excellent corrosion resistance is rendered ineffective if the primer does not adhere to the substrate well [25]. Figure 14, Figure 15 and Figure 16 illustrate the appearance of the pull-off adhesion test for all three primers. Table 5 shows the average pull-off adhesion results for each primer.

The dolly failure surfaces showed that PVB-ZC exhibits a combination of adhesive and cohesive failures. Its average pull-off adhesion is 4.79 MPa. On the other hand, EP has the lowest adhesion with an average of 3.20 MPa. Similar to PVB-ZC, the dolly failure surfaces of EP also show a mix of adhesive and cohesive coating failures. In contrast, PVB-ZO demonstrates the highest adhesion with a value of 5.93 MPa. Unlike the other two primers, PVB-ZO predominantly exhibits cohesive failures, indicating that the fracture occurs within the coating rather than at the coating-substrate interface. This characteristic highlights PVB’s superior adhesion and makes it more desirable as it reduces the risk of continuous peeling when the coating experiences repetitive damage.

## 4. Discussion: Primer Failure Mechanisms and Required Properties of Propeller Coatings

The results provide a basis for identification of the potential failure mechanisms and designing suitable propeller primers. It also helps to determine the most appropriate testing methods to assess the desired properties for propeller primers. The analysis focused on the nature of the coating system and its anticorrosive protection mechanism. Since propeller failure modes are complex, the desired properties for propeller coatings can only be determined through individual testing. Based on the overall scores, it can be concluded that PVB-ZO appears to be the most promising primer for designing propeller coatings. We expect that these findings will contribute valuable knowledge to the current propeller coating industry. 

### 4.1. Electrochemical Properties

#### 4.1.1. EIS and PDP Test

By comparing the EIS results of all three primers, it was found that the PVB-ZO primer demonstrates the highest barrier performance, indicated by the lowest water penetration. This improved performance could be attributed to the incorporation of ZnO in the primer formulation, which enhances its water resistance. The coating performance can be monitored by the change in shape of the Bode plot curves [19]. When an AC current is applied to a coating under good condition, a capacitor is used to describe the coating. This is also indicated in the Bode plot, where it exhibits a straight line with slope −1 at almost all frequencies for the capacitive region [18]. However, as the coating absorbs electrolyte, its deterioration can be observed by the development of resistive region (the curve towards the low frequency region becomes a plateau) on the Bode plot. This change was also observed in Figure 3, especially for PVB-ZC and EP. 

The development of the resistive region at low frequencies can indicate electrolyte diffusion and loss of coating–metal interface adhesion, which ultimately leads to corrosion underneath the coating. The low-frequency impedance modulus (|*Z*|_0.1Hz_) is a good indicator of the coating’s performance over time. Based on the |*Z*|_0.1Hz_ value of PVB-ZO (the low-frequency region is a representation of the coating–substrate interface [18,19]), its impedance value remains relatively constant over 100 days of immersion. In addition, the PDP test also confirms that PVB-ZO exhibits the highest level of substrate protection with a low electrolyte penetration rate through the primer film.

Despite its long-standing use and reputation for excellent field performance, the electrochemical test results of PVB-ZC have shown contradictions. It should be noted that the only difference between the field trial and the laboratory-based test is that the topcoat is not included in the lab test. Both the EIS and PDP tests show that PVB-ZC is more permeable compared to the other two primers when immersed in saltwater over time. As the immersion progresses, the *I_corr_* value increases, which is an indication of increasing electrolyte penetration in the coating. This observation can be attributed to the inhibitive nature of PVB-ZC. Inhibitive primers rely on the reaction between the corrosion inhibitor and the exposed metal surface to form a passive film, effectively shielding the substrate from corrosive ions. However, in order for the corrosion inhibitor to reach the exposed substrate, the primer has to have a certain degree of permeability for the corrosion inhibitor to diffuse within the coating first.

On the other hand, EP behaved differently compared to PVB-based primers. As the EIS results indicate, EP initially has the highest |*Z*|_0.1Hz_ but then it started to decrease continuously over time. This is because the electrolyte kept being absorbed by the coating through the pores and defects in the primer. The cause of these results is thought to be the fact that EP is formulated purely based on barrier protection (barrier pigments without any inhibitive pigments) rather than inhibitive mechanism. Since the coating is not 100% defect-free, electrolyte may first enter the tiny pinholes and pores and gradually form conductive pathways as the electrolyte continuously enters and leaves the coating [4]. As long as the electrolyte reaches the substrate, the corrosion process will start spontaneously. The PDP test showed that the protective ability of EP gradually decreased based on the decreasing *E_corr_* and increasing *I_corr_*.

#### 4.1.2. Artificially Scratched EIS Test

The scratched EIS test reveals the self-healing ability of the coating when it is subjected to damage. The application of damaged EIS test demonstrated PVB-ZC’s superiority over the standard EIS test in assessing its passivation ability. The |*Z*|_0.1Hz_ parameter of PVB-ZC exhibited a gradual increase over 100 days, surpassing the other two primers. This significant increase implies the formation of a passive film, which effectively serves as a protective shield preventing corrosion ions from reaching the substrate. 

However, the scratched EIS results reveal that PVB-ZO lacks the ability to self-heal. Under accelerated exposure conditions, its impedance gradually decreased. Additionally, ZnO gradually dissolves in cathodic areas, further affecting the performance. Also, no passivation ability was observed for EP as the coating is based on barrier protection solely.

#### 4.1.3. Electrolysis Test

**Figure 17:**
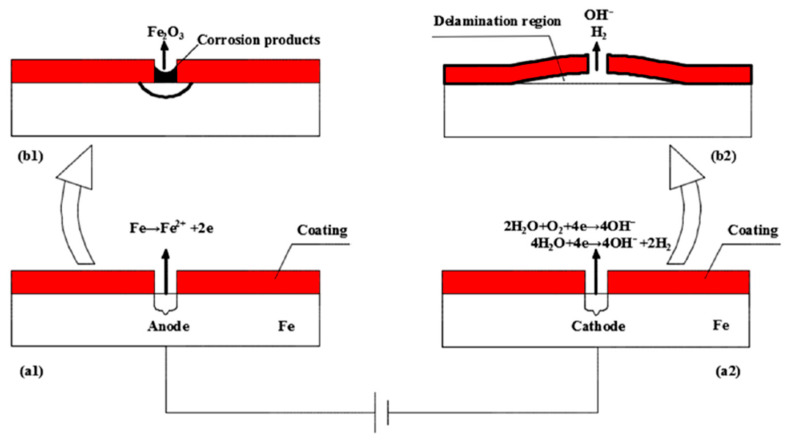
Schematic of the cathodic disbondment mechanism of pipeline under DC stray current. Dissolution of anode into metal ions (**a1**), forming metal oxide corrosion products on the anode (**b1**). Oxygen reduction reaction and hydrogen evolution occur at the cathode (**a2**), forming OH^-^ ions and hydrogen gas, causing coating delamination (**b2**). Reproduced from Ref. [26], Copyright (2019), with permission from MDPI.

The electrolysis test reveals that PVB-ZO develops a blackened colour when the current passes through the coated bronze plate. The detached black film, observed in the failed coating region, is likely due to the cathodic disbondment of scratched film. Figure 17 illustrates a stray current in a typical coated pipeline system, similar to the stray current corrosion observed in propellers except for the fact that the anode is not covered by a coating and a different substrate was used. The process of cathodic disbondment initiates at the anode, where a DC current flows and dissolves the anode to metal ions. These metal ions then react with H_2_O to form metal oxide, depositing on the corroded area of the anode. On the cathodic side, oxygen reduction and hydrogen evolution occur, producing H_2_ and OH^−^ ions. These species migrate along the interface between the coating and substrate, producing bubbles and forming a highly alkaline environment. This weakens the adhesion between the coating and substrate, causing cathodic disbondment of the coating from the substrate [26]. 

The cathodic disbondment mechanism of PVB-ZC is expected to be similar to that of PVB-ZO, considering the consistent sample preparation procedure and configuration. However, a notable distinction lies in the presence of zinc chromate in PVB-ZC, which operates through a different protective mechanism. The passivation ability of CrO_4_^2−^ ions may account for the formation of the silver-coloured area. However, the film formed is thin, with a thickness of less than 100 nm. Therefore, this observation may suggest the occurrence of reduction reactions and subsequent deposition of specific metal ions. A more comprehensive analysis is needed to investigate the nature of the silver-coloured film further.

On the other hand, the electrolysis result indicates that EP has the best performance at resisting cathodic disbondment around the artificial scratch. However, this could be due to the thickness of the EP being 200 µm, which is much thicker compared with PVB-based primers. As the bronze plate was prepared by brush, it is hard to control the primer at the same thickness. In addition, most epoxy primers only work at this level of thickness or greater for effective corrosion protection. 

### 4.2. Mechanical Properties

#### 4.2.1. Microhardness Test

The microhardness tests reveal that PVB-ZC is characterized by a soft nature, which is likely attributed to its film-forming mechanism. However, it is noteworthy that the microhardness of PVB-ZC surpasses that of PVB-ZO. This can be attributed to the incorporation of phosphoric acid as the activator for zinc chromate just prior to use. Upon activation, zinc chromate reacts with phosphoric acid, yielding a mixture of chromium in both cationic and anionic forms. Consequently, CrO_4_^2−^ becomes available for passivating the phosphate surface, thereby establishing a three-dimensional organic-metallic polymer coating [27]. The crosslinking reaction between phosphoric acid and zinc chromate may contribute to the increase in microhardness of PVB-ZC compared to PVB-ZO. This observation provides a plausible explanation for the higher microhardness of PVB-ZC in comparison to PVB-ZO.

Due to the different film-forming mechanisms of EP compared to PVB primers, its microhardness is much higher. The high crosslinking density gives a high microhardness. A chemical reaction occurs when the Epoxy resin crosslinks with polyamide, where the epoxide ring opens and bond with the curing agent to form crosslinks [25]. As a result, the three-dimensional crosslinking network gives rise to the high microhardness values.

#### 4.2.2. Conical Mandrel Bending Test

The conical mandrel bending test indicated that PVB-ZC initially exhibited flexibility prior to immersion but became brittle after immersion. This behaviour is similar to that of PVB-ZO, although it did not flake. The increased brittleness in both primers following immersion can be attributed to the permeability of the coatings. The pores allow for greater water absorption, leading to water accumulating at the metal–coating interface. Consequently, the wet adhesion is compromised to a greater extent, and more chromium ions are dissolved into the electrolyte, particularly in the absence of a topcoat. Due to the high flexibility and excellent adhesion of PVB-ZO, the conical mandrel bend tests prior to immersion showed no cracking. However, small cracks were found after immersion probably due to the dissolution of zinc oxide under alkaline conditions.

The conical mandrel bend test confirms that EP has higher microhardness but is more brittle than PVB-based primers. The high crosslinking density means that the crosslinked polymer chains are no longer flexible (chain mobility is restricted). However, the EP became more flexible after immersing in saltwater based on the conical mandrel bending test, probably due to water being able to plasticize the coatings to increase flexibility [4].

#### 4.2.3. Pull-Off Adhesion Test

PVB-ZO has the highest dry pull-off adhesion, which could be due to the excellent adhesion properties of PVB resin to metal substrates. However, with the same resin base, PVB-ZC has a lower dry pull-off adhesion than PVB-ZO. This might be due to the effect of zinc oxide particles to increase the dry adhesion. The observed failure modes in PVB-ZC adhesion test exhibited a combination of cohesive and adhesive failure and can be attributed to the formation of 3D organic–metallic polymer matrix and possibly the low vol.% of the solid content compared with PVB-ZO, which reduced the cohesive force in the coating. 

EP has the lowest pull-off adhesion than the other two primers and the failure dolly surfaces showed mostly adhesive failure. The high microhardness and brittleness of EP might cause the low adhesion and adhesive failure. The low dry pull-off adhesion also serves as a complimentary result to explain the peeling observed for the dry conical mandrel bend test.

### 4.3. Analysis Conclusion

Based on the results of PVB-ZC, it was found its porous nature is intentionally designed to facilitate the diffusion of CrO_4_^2−^ ions, allowing the formation of a passivation film on the exposed metal surface. However, as the primer remains submerged over time, the zinc chromate content gradually depletes. Without sufficient zinc chromate, the metal becomes susceptible to corrosion again [28]. In practical field applications, a topcoat is promptly applied over the PVB-ZC primer to serve multiple functions. Firstly, it reduces the adhesion of microorganisms to the propeller by employing a foul-release mechanism. Additionally, the resilience of the topcoat allows it to recover from cavitation damage. Furthermore, it plays a crucial role in restricting the amount of water penetration through the primer, thereby preventing excessive leaching of CrO_4_^2−^ ions. 

EP is more susceptible to mechanical damage due to its brittleness and low adhesion to the substrate. The flaking of the primer will be a continuous process once the primer is damaged by mechanical factors. A barrier protection-based epoxy primer is vulnerable to cavitation damage and electrochemical corrosion as there is no inhibitive pigment that can suppress/slow down corrosion as the electrolyte being absorbed by the primer. 

In summary, the most desired properties for an anticorrosive propeller primer are (1) to be able to self-heal after the coating is damaged, (2) to be flexible to avoid cracking and flaking, (3) to be able to resist cathodic disbondment (electrolysis), and (4) adequate primer–substrate adhesion. Furthermore, the primer matrix should be porous to a certain degree if the protection mechanism is inhibitive based. On the other hand, if the primer relies solely on the barrier protection mechanism, the primer matrix should be denser to reduce water permeability but with a certain level of flexibility. Overall, PVB-ZO is more suitable than EP for designing propeller coatings due to its flexibility, excellent dry adhesion, and high barrier performance over an extended period. However, PVB-ZO did not exhibit any anticorrosive properties as demonstrated by the electrochemical tests, which means that the corrosion process cannot be terminated or slowed down once it occurs. Introducing a self-healing capability could be one of the promising options for further optimization of PVB-ZO for propeller applications. 

## 5. Conclusions

Coatings for marine propellers are subjected to complicated failure forms. This work studies the required properties for designing and selecting a propeller coating. A series of electrochemical, corrosion, and mechanical tests were performed to explore the desired properties for marine propeller coatings. The results indicate that the following properties are important for marine propeller coatings:

(1) Excellent adhesion between the primer and the substrate to ensure the primer is intact on the substrate even when water is absorbed into the coating;
(2) Flexibility to minimize cracking and to avoid continuous flaking upon a coating damage;
(3) Resistance to electrolysis/cathodic disbondment and high alkalinity in the cathodic area;
(4) Self-healing after the coating is damaged. This is often achieved by controllable release of a corrosion inhibitive to slow down the corrosion when the coating is damaged.

By assessing the PVB-ZO and EP primer against the criteria described above, we conclude that PVB-ZO primer is more suitable for the design of marine propeller coatings. It is believed that developing a self-healing PVB primer could be one of the viable options for enhancing the performance of marine propeller coatings.

## Figures and Tables

**Table 1:** The nature of the primers being tested.

Primer	Coating System	Main Active Component
PVB-ZC	2K PVB	PVB base, zinc chromate, talc Hardener: phosphoric acid
PVB-ZO	1K PVB	PVB base, ZnO
EP	2K Epoxy	Epoxy base, talc, mica, barium sulfate, titanium dioxide Hardener: polyamide

**Table 2:** Substrate type used for each test.

Test	Substrate	Dimensions (mm)
Electrochemical Impedance Spectroscopy	C95800 Al Bronze	100 × 100 × 3.0
Potentiodynamic Polarization Test	C95800 Al Bronze	100 × 100 × 3.0
Microhardness	Al 5005	100 × 150 × 1.5
Mandrel Bend	Al 1100	100 × 150 × 0.8
Pull-off Adhesion	C95800 Al Bronze	100 × 100 × 3.0
Electrolysis	C95800 Al Bronze	200 × 200 × 3.0

**Table 3:** The average dry film thickness (DFT) of each primer for each test.

Test	DFT (µm) ± STDEV
Electrochemical Impedance Spectroscopy	PVB-ZC: 102.7 ± 3.6
	PVB-ZO: 107.3 ± 3.0
	EP: 99.3 ± 6.1
Potentiodynamic Polarization Test	PVB-ZC: 116.3 ± 8.0
	PVB-ZO: 111.3 ± 14.7
	EP: 157.6 ± 13.5
Microhardness	PVB-ZC: 325.9 ± 16.4
	PVB-ZO: 309.0 ± 14.4
	EP: 314.7 ± 19.8
Mandrel Bend	PVB-ZC: 94.4 ± 4.2
	PVB-ZO: 96.0 ± 6.6
	EP: 107.6 ± 10.9
Pull-off Adhesion	PVB-ZC: 141.9 ± 8.6
	PVB-ZO: 150.7 ± 9.2
	EP: 141.9 ± 12.7
Electrolysis	PVB-ZC: 36.4 ± 4.4
	PVB-ZO: 43.5 ± 7.1
	EP: 240.0 ± 47.9

**Table 4:** Microhardness (HV0.1) results of primers.

Primers	Average Hardness (HV0.1)	STDEV
PVB-ZC	3.90	0.20
PVB-ZO	1.60	0.10
EP	17.76	2.61

**Table 5:** Pull-off adhesion results.

Primers	Average MPa ± STDV
PVB-ZC	4.79 ± 0.28
PVB-ZO	5.93 ± 0.26
EP	3.20 ± 0.57

## Data Availability

Data are contained within the article.
